# Correlation between Carboxyhemoglobin Levels Measured by Blood Gas Analysis and by Multiwave Pulse Oximetry

**DOI:** 10.3390/jpm14020168

**Published:** 2024-01-31

**Authors:** Jisu Yu, Juncheol Lee, Yongil Cho, Jaehoon Oh, Hyunggoo Kang, Tae Ho Lim, Byuk Sung Ko

**Affiliations:** Department of Emergency Medicine, College of Medicine, Hanyang University, Seoul 04763, Republic of Korea; yulisu20000@hanmail.net (J.Y.); doldoly@hanyang.ac.kr (J.L.); joeguy@hanmail.net (Y.C.); ojjai@hanmail.net (J.O.); emer0905@hanmail.net (H.K.); erthim@hanyang.ac.kr (T.H.L.)

**Keywords:** emergency department, COHb (carboxyhemoglobin), multiwave pulse oximetry, sensitivity, specificity

## Abstract

Carbon monoxide (CO) poisoning is difficult to diagnose owing to its nonspecific symptoms. Multiwave pulse oximetry can be used to quickly screen patients for CO poisoning. However, few studies have analyzed patients with CO poisoning who presented to the emergency department (ED). The primary aim of our study was to determine the correlation between COHb levels measured in blood gas analysis and COHb levels measured in multiwave pulse oximetry. Secondary aims were the sensitivity and specificity of the COHb level cutoff value using multiwave pulse oximetry to predict a 25% COHb level in blood gas analysis. This single-center retrospective observational study included patients with CO poisoning who visited the ED of a university-affiliated hospital in Seoul, Korea between July 2021 and June 2023. COHb poisoning was determined using blood gas analysis and multiwave pulse oximetry. The correlation of COHb levels between the two tests was evaluated using correlation analysis. The area under the receiver operating characteristic curve (AUC) of multiwave pulse oximetry was calculated to predict COHb levels from the blood gas analysis. The optimal cutoff values, sensitivity, and specificity of COHb were determined. A total of 224 patients who had COHb levels measured using both multiwave pulse oximetry and blood gas analysis were included in the analysis. In the correlation analysis, COHb showed a high positive correlation with COHb measured using blood gas analysis (Spearman correlation coefficient = 0.86, *p* < 0.001). The AUC of COHb measured by multiwave pulse oximetry to predict 25% of the COHb level (which can be an indication of hyperbaric oxygen treatment) measured by blood gas analysis was 0.916. When the COHb levels measured with multiwave pulse oximetry were 20% the sensitivity was 81% and the specificity was 83%, and when the COHb levels were 25% the sensitivity was 50% and the specificity was 95%. The COHb value measured using multiwave pulse oximetry blood gas analysis showed a high correlation. However, additional research using large-scale studies is required for validation.

## 1. Introduction

Carbon Monoxide (CO) is produced due to incomplete combustion of fuel. The main sources of CO are inhalation of smoke from fires, poorly functioning heating systems, poorly ventilated fuel-burning appliances, automobiles operating in poorly ventilated areas, outdoor exposure to motorboat exhaust, and large amounts of CO emissions [1]. An increase in CO poisoning has been reported to occur in the immediate aftermath of electrical power outages, leading to increased use of portable, gasoline-powered generators. Generator misplacement indoors or proximate to home ventilation intake systems causes CO exposure and occasional fatalities. CO, a colorless, odorless, and non-irritating gas, is generated through the incomplete combustion of hydrocarbons, rendering the diagnosis of CO poisoning challenging owing to its nonspecific symptoms which include headache, confusion, nausea, dizziness, chest pain, shortness of breath, and seizures [2,3,4,5,6,7]. Intentional CO poisoning accounts for two-thirds of deaths, and inadvertent, non-fire-related CO poisoning causes the rest [8]. In contrast with intentional poisoning, unintended poisoning demonstrates both seasonal and regional variation, and occurs more frequently during the winter months in cold climates, most commonly from faulty furnaces [9]. CO is a leading cause of poisoning and is associated with high morbidity and mortality rates [10,11,12]. Data from the United States show approximately 20,000 exposure cases and 439 deaths each year, with a particular focus on unintentional exposure and non-fire-related incidents due to CO poisoning [13,14]. It is the third leading cause of unintentional poisoning death in the United States [2]. However, registry trials indicate significantly higher figures than those officially reported, with approximately 50,000 visits to ED annually, which accounts for 0.05% of all patients. In the United States, CO poisoning was responsible for approximately 450 deaths every year from 1999 to 2004 and an estimated annual average of about 15,200 ED visits from 2001 to 2003 [2,3,14]. In France, approximately 6,000 patients receive treatment each year for suspected or confirmed CO poisoning, with 2,500 patients requiring hospital admission, and 300 deaths. Data from other countries are limited, but higher incidence rates than that in the United States and Europe are anticipated [2,3]. Analysis of aggregated national data from the United States supports an overall mortality of 1 to 3 percent, with a mortality rate that is higher for intentional poisoning than for inadvertent exposure [11]. There are approximately 1000 to 1300 deaths from CO poisoning annually [8,10]. 

CO has an affinity for hemoglobin that is approximately 220 times greater than that of oxygen, which hinders oxygen delivery to tissues [4,5,8]. Furthermore, CO binds to myoglobin, exacerbates hypoxia in the cardiac muscles, and triggers blood clotting and neutrophil activation. Approximately 10 to 15 percent of CO is extravascular and bound to molecules such as myoglobin and cytochromes, resulting in impairment of oxidative phosphorylation at the mitochondrial level [15,16]. The half-life of CO bound to these molecules is longer than that of COHb. The importance of these non-hemoglobin-mediated effects has been best documented in the heart, where mitochondrial dysfunction due to CO can produce myocardial stunning despite adequate oxygen delivery [17]. Additionally, it initiates the generation of free radicals, leading to acute tissue damage in the brain, heart, and other organs [4,8,18]. Although there is no well-defined diagnostic criteria, survivors of severe acute poisoning have reported delayed neuropsychiatric sequelae characterized by symptoms such as memory loss, impaired concentration or language abilities, mood changes including depression, and symptoms resembling Parkinson's disease [4,5,8,12,18,19,20,21]. These sequelae are reported to arise 3 to 240 days after apparent recovery, generally occurring within 20 days of CO poisoning [22,23,24]. The mechanism of the sequelae is incompletely understood, but it probably involves lipid peroxidation by reactive oxygen species generated by xanthine oxidase. Xanthine oxidase is produced in situ from xanthine dehydrogenase via enzymes released by white blood cells that adhere to damaged endothelial cells [25,26]. During recovery from CO exposure, events analogous to ischemia-reperfusion injury and exposure to hyperoxia may exacerbate the initial oxidative damage [27]. Consequently, CO poisoning requires immediate treatment because of its high morbidity and mortality rates, necessitating rapid and rational screening.

Carboxyhemoglobin (COHb) levels do not comprehensively indicate the severity of CO poisoning. Nevertheless, they serve as foundational factors for assessing the necessity of hyperbaric oxygen therapy. COHb percentage measured by laboratory analysis of venous or arterial blood samples is currently the standard method used to detect and quantify CO poisoning [28,29]. However, conducting a COHb test by laboratory analysis of venous or arterial blood samples may not be immediately available in all hospitals. In addition, in a prehospital setting, a blood sample may not always be available. Consequently, many patients with CO poisoning may be overlooked and misdiagnosed [4]. Traditional pulse oximetry uses only two different wavelengths of light, making it impossible to accurately determine COHb or methemoglobin levels, potentially leading to erroneous results. Recent technological advancements have enabled noninvasive measurement of COHb through multiwave pulse oximetry. Multiwave pulse oximeters utilize eight distinct wavelengths to address these limitations [28,30,31]. Measuring COHb levels in the prehospital setting using multiwave pulse oximetry could be simply used as a screening tool whether CO poisoning is present at all and in the decision-making process regarding the transfer of patients to specialized medical facilities for CO poisoning. However, few studies have analyzed the correlation between COHb levels measured using standard blood gas analysis and multiwave pulse oximetry in ED patients.

This study aimed to evaluate the correlation between COHb levels measured using standard blood gas analysis and multiwave pulse oximetry. In addition, the cutoff value measured by multiwave pulse oximetry corresponding to the COHb level in the blood gas analysis was determined.

## 2. Materials and Methods

### 2.1. Study design and Population

This retrospective, observational cohort study was conducted between July 2021 and June 2023. This study was conducted on carbon monoxide poisoning in adults aged 18 or older who visited the ED of a university hospital in Seoul. Our hospital treats approximately 150 patients with CO poisoning each year. The study patients were initially identified via electronic computerized medical records by using a hospital discharge diagnosis search for “Toxic effect of carbon monoxide (International Classification of Diseases-10 code T58)” from July 2021 to June 2023. A diagnosis of acute CO poisoning was based on history-taking by the emergency physicians on duty and a COHb concentration > 5% (>10% in current smokers) in blood gas analysis [32].

### 2.2. COHb Measurement and Treatment

We compared the COHb levels from blood gas analysis (arterial or venous) and multiwave pulse oximetry. COHb levels were measured using a point-of-care blood gas analyzer (GEM Premier 5000 with iQM [Bedford, MA, USA]) or a multiwave pulse oximeter (MASIMO Rad-97 Pulse CO-Oximeter [Irvine, CA, USA]). For blood gas analysis, either arterial or venous blood was used. The Rad-97 Pulse CO-Oximetry technology is governed by the following principles: Oxyhemoglobin (oxygenated blood), deoxyhemoglobin (non-oxygenated blood), carboxyhemoglobin (blood with carbon monoxide content), methemoglobin (blood with oxidized hemoglobin), and blood plasma constituents differ in their absorption of visible and infrared light (using spectrophotometry). The amount of arterial blood in tissue changes with pulse (photoplethysmography). Therefore, the amount of light absorbed by the varying quantities of arterial blood changes as well. The Rad-97 utilizes a sensor with various light-emitting diodes (LEDs) that pass light through the site to a diode (detector). Signal data are obtained by passing various visible and infrared lights (LEDs, 500 to 1400 nm) through a capillary bed (for example, a fingertip, a hand, or a foot) and measuring changes in light absorption during the blood pulsatile cycle. This information may be useful to clinicians. The maximum radiant power of the strongest light is rated at ≤25 mW. The detector receives the light, converts it into an electronic signal, and sends it to the Rad-97 for calculation. The data of patients admitted to the ED were extracted through chart review. All patients received standard treatment followed by hyperbaric oxygen therapy and oxygen delivery via a face mask with a reservoir bag. Hyperbaric oxygen therapy was administered if the patients showed signs of severe intoxication (e.g., unconsciousness, neurological signs, cardiovascular dysfunction, or severe acidosis) or if the COHb was greater than 25%. Hyperbaric oxygen treatment was performed in a monoplace hyperbaric oxygen chamber. One session of hyperbaric oxygen therapy lasted a total of two hours, targeting a pressure of 2.5 standard atmospheres. It was performed once a day, and the number of times was determined at the discretion of the attending physician. Patients who did not undergo multiwave pulse oximetry were excluded. The Institutional Review Board of our hospital approved this study and waived the requirement for informed consent due to its retrospective nature.

### 2.3. Variables and Outcome

Demographic characteristics, vital signs, underlying diseases, symptoms, signs, and blood test variables were collected through chart review. Our study population included adults aged > 18 years who visited the ED due to CO poisoning between July 2021 and June 2023. Patients who underwent blood gas analysis and multiwave pulse oximetry were included. The primary outcome was the correlation between COHb levels from blood gas analysis and those from multiwave pulse oximetry. The secondary outcomes were the sensitivity and specificity of the COHb level cutoff value using multiwave pulse oximetry to predict a 25% COHb level in blood gas analysis.

### 2.4. Statistical Analysis

Continuous variables were analyzed as means ± standard deviation or median with interquartile range (IQR.), and categorical variables were analyzed as absolute or relative frequencies. Student’s *t* tests or Mann Whitney U tests were used to compare continuous variables, and the chi-squared test or Fisher’s exact test was used for categorical variables.

Correlation between COHb levels measured by blood gas analysis and multiwave pulse oximetry was compared using Spearman’s correlation analysis. A scatterplot was drawn for COHb values from multiwave pulse oximetry and COHb values from blood gas levels. The area under the curve (AUC) of multiwave pulse oximetry for predicting COHb levels from blood gas analysis was calculated. The optimal cutoff values of COHb as measured by multiwave pulse oximetry maximizing sensitivity and specificity to predict COHb with blood gas analysis were determined using the Youden index. The sensitivity, specificity, positive predictive value (PPV), and negative predictive value (NPV) were determined. A two-tailed *p* value < 0.05 was considered statistically significant. All statistical analyses were performed using SPSS version 18 software (IBM Inc., Chicago, IL, USA).

## 3. Results

Amongst the 304 patients screened through chart review from July 2021 to August 2023, 80 patients were excluded because they did not undergo multiwave pulse oximetry, and their measurements were unavailable. A total of 224 patients who underwent both multiwave pulse oximetry and blood gas analyses were included for analysis.

Baseline demographic and clinical characteristics of the patients are presented in Table 1. The median age was 37 years (IQR, 28–51 years). Of the total patients, 146 were male (61.6%). The median systolic blood pressure was 126 mm Hg (IQR, 114–141). The median diastolic blood pressure was 80 mm Hg (IQR, 70–89). The median pulse rate per minute was 94 (IQR, 78–108). The median respiratory rate per minute was 18 (IQR, 18–20). Hypertension was reported in 37 patients (15.6%), and diabetes mellitus in 14 patients (5.9%), and 138 patients (58.2%) were smokers. The proportion of patients with hypertension and diabetes mellitus by age and gender are shown in the Supplementary Figures S1–S4. The prevalence of hypertension tended to increase with age. 1.8% of those in their 30s, 12.2% of those in their 40s, 10% of those in their 50s, and 15.4% of those in their 60s had high blood pressure as an underlying disease. The proportion of patients with diabetes, an underlying disease, tends to increase with age, reaching 12.2% in those in their 40s, 10% in their 50s, 15.4% in their 60s, and 15.4% in their 70s. The prevalence of hypertension by gender was 6.5% for men and 4.6% for women. The prevalence of diabetes was 6.5% in men and 4.6% in women. We defined smokers as current smokers. The remaining 86 (41.8%) patients did not smoke or had smoked in the past. Seven patients (3.1%) had coronary artery disease as an underlying disease. One patient (0.4%) had coronary artery disease as an underlying disease. Loss of consciousness occurred in 128 patients (54%). Dizziness was observed in 99 patients (41.8%). Fifty patients (21.1%) complained of chest pain. Ninety-two (41.1%) patients complained of headache. Dyspnea was observed in 63 patients (28.1%). Of the total patients, 167 (71.9%) were poisoned with carbon monoxide for the purpose of attempting suicide. 

In laboratory tests, the median COHb level in blood gas analysis was 10.1% (IQR, 4.6–19.5). The median COHb level in multiwave pulse oximetry was 11% (IQR, 3–19). The median Troponin I level was 0.01 ng/mdL (IQR, 1–3.1). The median white blood cell (WBC) level was 10.3 10^3^/mm^3^ (IQR, 7.5–14.2). The median hemoglobin level was 14.6 g/dL (IQR, 13.2–15.8). The median platelet level was 243 (IQR, 208–295). The median creatine kinase (CK) level was 140 (IQR, 82–301). The median BUN level was 13.6 mg/dL (IQR, 10.4–17). The median creatinine level was 0.8 mg/dL (IQR, 0.6–1). The median creatine kinase myocardial band (CK MB) level was 1.3 ng/mL (IQR, 1–3.1). The median Troponin I level was 0.01 ng/mdL (IQR, 1–3.1). The median brain natriuretic peptide (BNP) level was 12 pg/mL (IQR, 6–43). The median CRP level was 0.3 mg/dL (IQR, 0.3–0.7). (Table 1).

**Figure 1:**
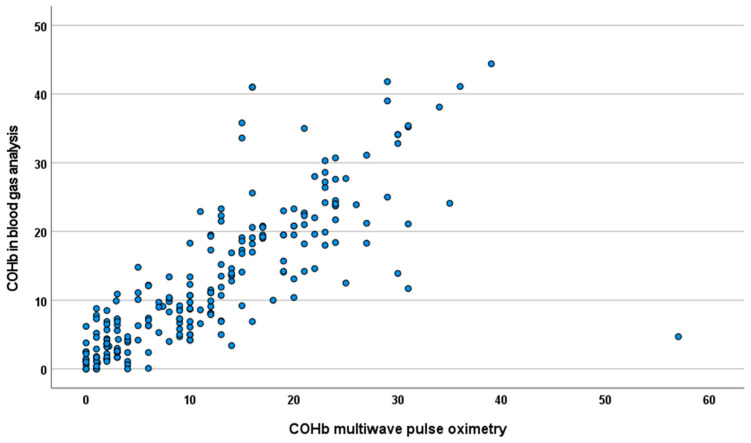
Scatter plot of COHb values of multiwave pulse oximetry and COHb values of blood gas analysis. COHb: carboxyhemoglobin.

**Figure 2:**
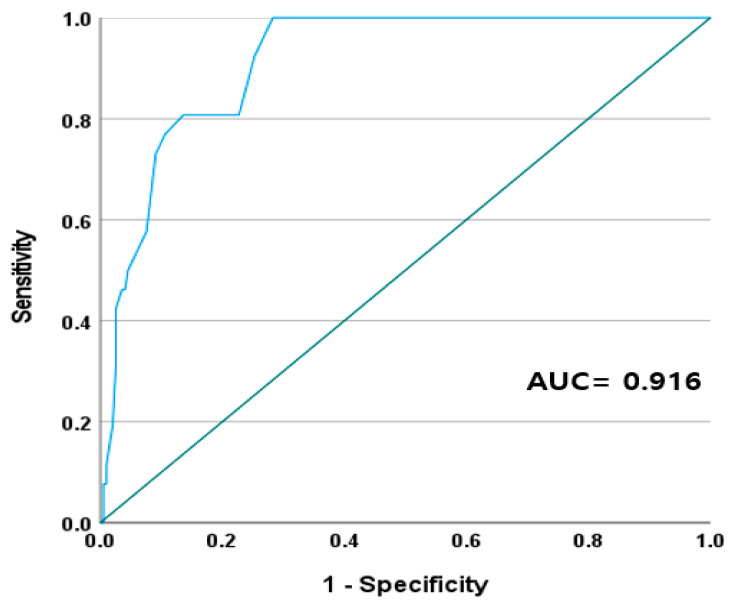
Receiver operating characteristic curves of multiwave pulse oximetry to predict 25% of the COHb level measured by blood gas analysis. COHb: carboxyhemoglobin. Green line represents the reference. Blue line represents the performance of multiwave pulse oximetry.

Figure 1 shows a scatter plot comparing the COHb levels obtained from multiwave pulse oximetry with those obtained from blood gas analysis. In the correlation analysis, the COHb measured using multiwave pulse oximetry exhibited a strong positive correlation with the COHb measured using blood gas analysis (Spearman’s correlation coefficient = 0.86, *p* < 0.001). The AUC of the COHb measured using multiwave pulse oximetry for predicting 25% of the COHb levels measured using blood gas analysis was 0.916 (Figure 2). 

The diagnostic performance of the COHb levels determined by multiwave pulse oximetry in predicting 25% of the COHb levels from blood gas analysis is shown in Table 2. The optimal cutoff values for the COHb levels in multiwave pulse oximetry to predict the COHb levels in 25% of the blood gas analyses are shown in Table 2. When the COHb levels were ≥15%, the sensitivity was 100% and specificity was 72%; for COHb levels ≥20% the sensitivity was 81% and specificity was 83%; and for COHb levels ≥25% the sensitivity was 50% and the specificity was 95%. As the cutoff value increased from 15% to 25%, the sensitivity decreased but the specificity increased. The PPV values for the 15%, 20%, and 25% COHb cutoff values by multiwave pulse oximetry were 32%, 39%, and 59%, respectively. The NPV values were 100%, 97%, and 94%, respectively (Table 2).

## 4. Discussion

The results of this study indicated a strong positive correlation between COHb levels measured by multiwave pulse oximetry and by blood gas analysis. This suggests that multiwave pulse oximetry can be a valuable tool for the rapid assessment of COHb levels in patients with suspected CO poisoning. Additionally, we believe that the sensitivity and specificity of the specific cutoff values determined in this study could be helpful in clinical practice for estimating blood COHb levels and deciding whether to transfer patients to a CO poisoning specialized treatment center.

The noninvasive pulse oximetry test is not a recent technology and is a topic already covered in the literature [33,34,35]. One notable finding in this study is the optimal cutoff value for COHb levels as measured by multiwave pulse oximetry, which helps physicians determine whether transfer to a specialized CO poisoning treatment center is necessary. We present three multiwave pulse oximetry COHb cutoff values. Hyperbaric oxygen therapy should not be based solely on blood COHb levels but should also include the presence or absence of evidence of neurological abnormalities or organ ischemia. When the cutoff value was set at 20% or 25%, the specificity increased but the sensitivity decreased, so there are cases where diagnosis is not made even if ischemia is severe, so caution is required when applying it to patients in clinical practice. It is also important to acknowledge the need for further prospective and large-scale studies to validate these findings.

Previous studies on multiwave pulse oximetry have produced diverse results. Barker et al. in a study on ten healthy volunteers compared measurements of COHb levels using multiwave pulse oximetry and blood oximetry [28]. In that study, the authors artificially elevated COHB levels by up to 15% to determine the degree of correlation between the two devices and to analyze the feasibility of noninvasive multiwave pulse oximetry. Another study analyzing the accuracy between multiwave pulse oximetry and blood COHb oximetry measurements in 1587 patients in the ED reported that noninvasive pulse oximetry measured COHb levels with acceptable precision [14]. However, that study included only 17 patients with CO poisoning, and analyzing the relationship between the two devices was difficult. Sebbaneet et al. reported that multiwave pulse oximetry cannot replace standard blood COHb oximetry given its moderate correlation and low precision [13]. In a recent study by Touger et al. involving 120 patients, the limits of agreement exceeded those selected as clinically acceptable [29]. These previous studies had relatively small sample sizes, and the criteria for patient inclusion were not similar to those in our data. Moreover, a median COHb level of 2.3% in a potentially CO-exposed patient population appears remarkably low [29]. These factors raise questions regarding the precision and accuracy of the measurement technique and the diversity in the studied patient populations.

A strength of our study is that we analyzed the correlations of COHb levels between multiwave pulse oximetry and blood gas analysis in the largest number of patients with suspected CO poisoning to date. Compared with previous research, the strength of this study lies in its ability to identify a specific COHb threshold, allowing clinicians to make informed decisions regarding transfer to a specialized treatment center for CO poisoning. This is particularly relevant in settings in which blood gas analysis may not be readily available or practical. The rapid identification of patients who require transfer to specialized CO poisoning treatment center is of paramount importance, as it not only aids in optimizing patient outcomes, but also plays a pivotal role in reducing the morbidity and mortality associated with CO poisoning. Therefore, the contributions of this study are invaluable and offer a more accessible and efficient method of managing suspected CO poisoning cases.

However, this study has several limitations. First, this was a single-center retrospective analysis, with a small sample size. Hence, the findings of our study cannot be generalized to other medical institutions. The potential impact of bias is more pronounced than in multicenter analyses involving larger populations. Given that false-negative readings can have serious medical implications, it is imperative that the findings of this study are validated in a larger multicenter cohort of patients with CO poisoning. Second, variations in sensor placement, attachment times, and healthcare providers responsible for sensor applications could potentially have introduced bias into our results. Nonetheless, considering that the clip was simply placed on the finger according to the manufacturer's instructions, the likelihood of significant variation appears to be minimal. Third, our study cohort primarily included Korean patients. Therefore, our findings cannot be extrapolated to patients of other races or nationalities. Last, the primary aim of our study was to determine the correlation between COHb levels measured in blood gas analysis and COHb levels measured in multiwave pulse oximetry. However, due to the constraints imposed by our limited sample size, we were unable to analyze cases in which patients exhibited false-negative results in multiwave pulse oximetry for COHb levels despite suffering from severe CO poisoning. This issue underscores the need for well-designed, large-scale prospective studies to address this gap in the literature.

## 5. Conclusions

In conclusion, this study demonstrated a strong positive correlation between COHb levels measured using multiwave pulse oximetry and blood gas analysis in patients with suspected CO poisoning. It is important to identify multiple cutoff values for multiwave pulse oximetry with high diagnostic performance to help physicians determine whether transfer to a specialized CO poisoning treatment center is necessary. Multiwave pulse oximetry shows promise as a valuable screening tool for initial assessment of CO poisoning. However, validation studies in larger cohorts are needed to identify the potential influencing factors on multiwave pulse oximetry results, and confirm its clinical utility.

## Figures and Tables

**Table 1:** Characteristics of study participants.

Patients Included (*n* = 224)	*n* (%) or Median (IQR), as Appropriate
Age, y, median (IQR)	37 (28-51)
Male, No. (%)	146 (61.6)
Systolic blood pressure, mm Hg, median (IQR)	126 (114–141)
Diastolic blood pressure, mm Hg, median (IQR)	80 (70–89)
Pulse rate, beats/min, median (IQR)	94 (78–108)
Respiratory rate,/min, median (IQR)	18 (18–20)
Comorbidities	
Hypertension, No. (%)	37 (15.6)
Diabetes mellitus, No. (%)	14 (5.9)
Smokers, No. (%)	138 (58.2)
CAD, No. (%)	7 (3.1)
Stroke, No. (%)	1 (0.4)
Loss of conscious, No. (%)	128 (54)
Dizziness, No. (%)	99 (41.8)
Chest pain, No. (%)	50 (21.1)
Headache, No. (%)	92 (41.1)
Dyspnea, No. (%)	63 (28.1)
Intentional exposure, No. (%)	161 (71.9)
COHb, blood gas analysis (%)	10.1 (4.6–19.5)
COHb, multiwave pulse oximetry (%)	11 (3–19)
WBC (10^3^/mm^3^)	10.3 (7.5–14.2)
Hemoglobin (g/dL)	14.6 (13.2–15.8)
Platlelets (10^3^/mm^3^)	243 (208–295)
CK (U/L)	140 (82–301)
Lactate (mmol/L)	2 (1–3.4)
BUN (mg/dL)	13.6 (10.4–17)
Creatinine (mg/dL)	0.8 (0.6–1)
CKMB (ng/mL)	1.3 (1–3.1)
Troponin I (ng/mL)	0.01 (0.01–0.05)
BNP (pg/mL)	12 (6–43)
CRP (mg/dL)	0.3 (0.3–0.7)

BNP: brain natriuretic peptide; BUN: blood urea nitrogen; CAD: coronary artery disease; COHb: carboxyhemoglobin; CK: creatine kinase; CK-MB: creatine kinase myocardial band; CRP: C-reactive protein; IQR: interquartile range; WBC: white blood cell.

**Table 2:** Diagnostic performance of COHb levels by multiwave pulse oximetry to predict 25% COHb levels from blood gas analysis.

COHb Level by Multiwave Pulse Oximetry, %	Sensitivity	Specificity	PPV	NPV
≥15	100 (%)	72 (%)	32 (%)	100 (%)
≥20	81 (%)	83 (%)	39 (%)	97 (%)
≥25	50 (%)	95 (%)	59 (%)	94 (%)

COHb: carboxyhemoglobin; NPV: negative predictive value; PPV: positive predictive value.

## Data Availability

Additional data are available on request from the corresponding author.

## Supplementary Materials

The following supporting information can be downloaded at: https://www.mdpi.com/article/10.3390/jpm14020168/s1, Figure S1: Proportion of patients with hypertension by age group; Figure S2. Proportion of patients with diabetes mellitus by age group; Figure S3. Proportion of patients with hypertension by gender; Figure S4. Proportion of patients with diabetes mellitus by gender.
