# Supplementary material for: Correlation between Carboxyhemoglobin Levels Measured by Blood Gas Analysis and by Multiwave Pulse Oximetry
Source: J Pers Med. 2024 Jan 31;14(2):168. doi: 10.3390/jpm14020168 (PMC10890311; doi:10.3390/jpm14020168)
Supplement: Supplementary file 1 [file jpm-14-00168-s001.zip › jpm-2805601-supplementary.pdf]

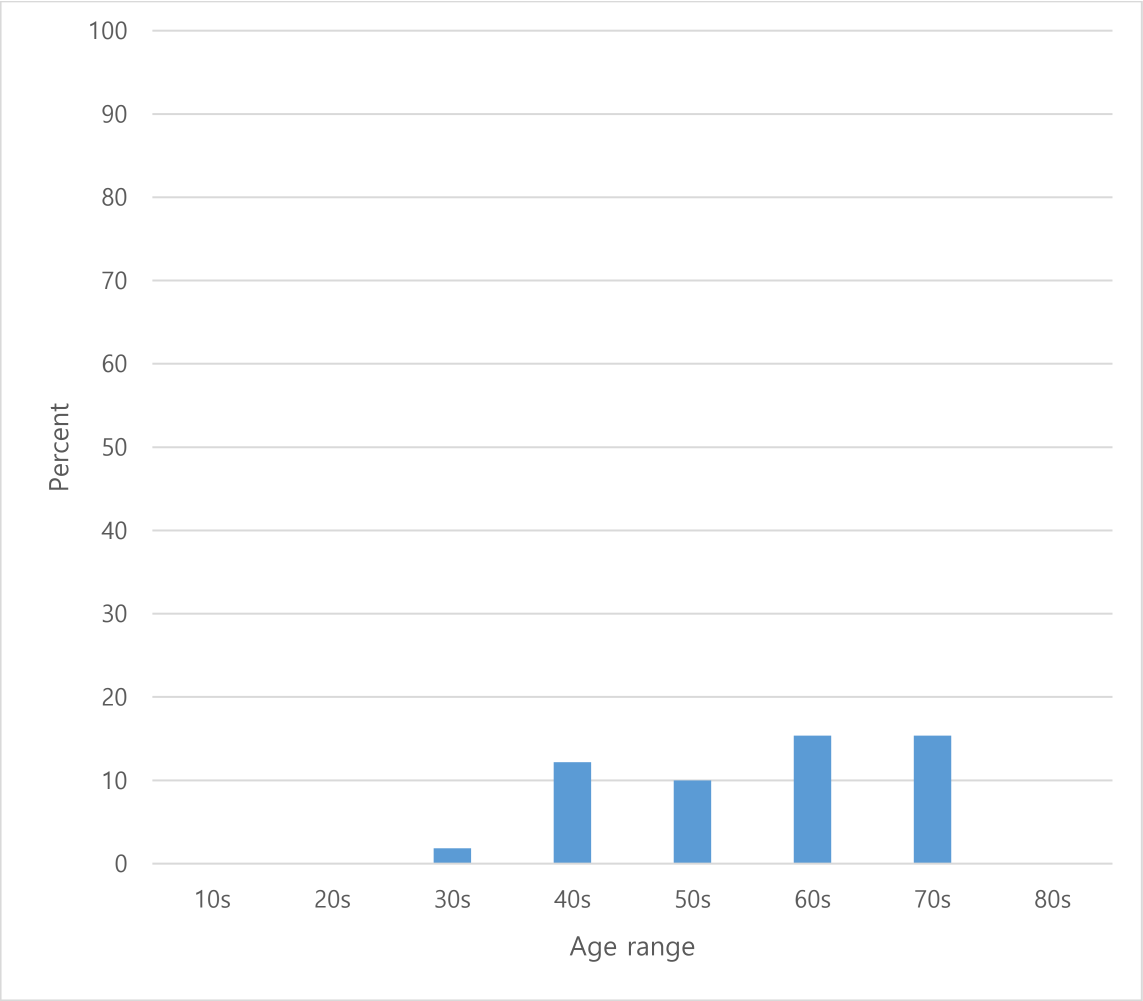

Figure S1. Proportion of patients with hypertension by age group.

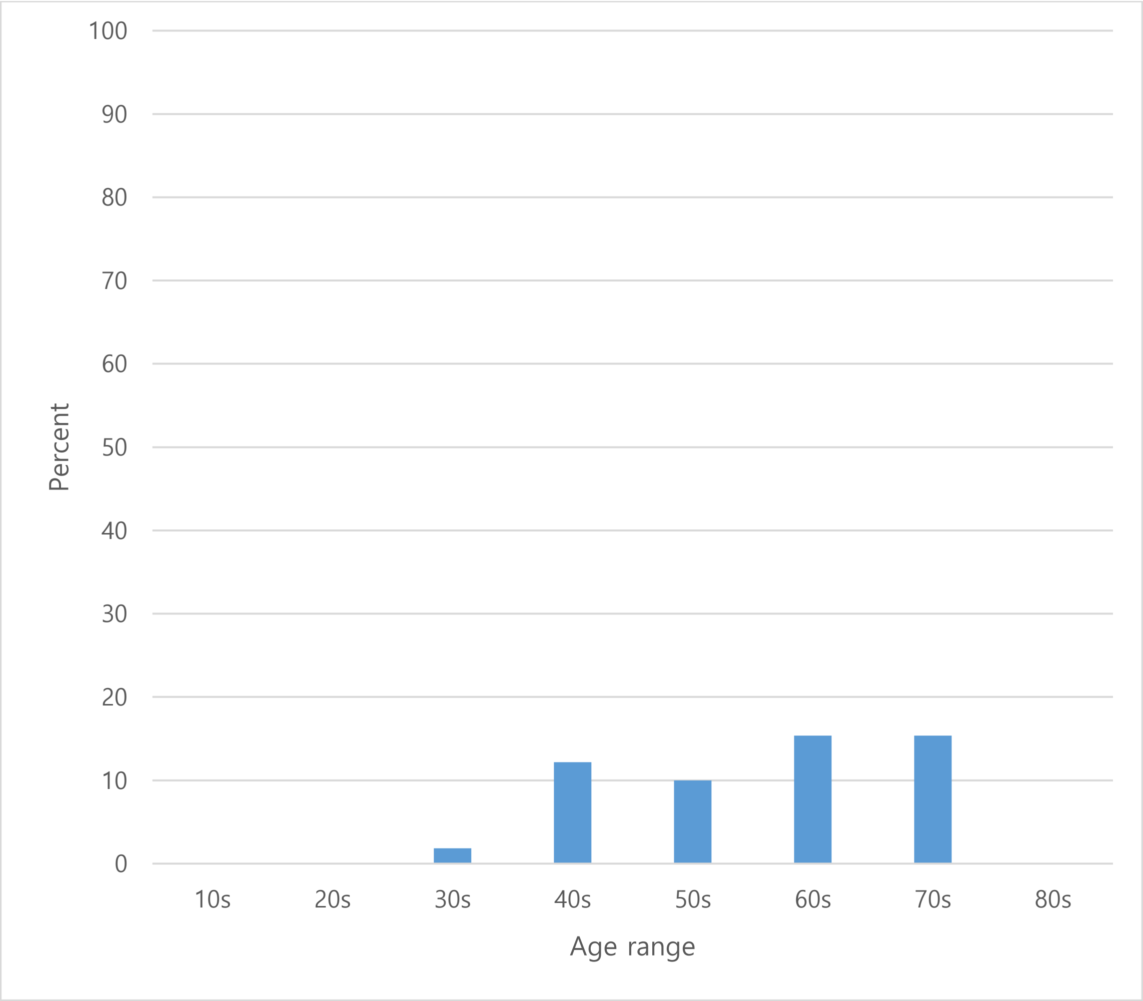

Figure S2. Proportion of patients with diabetes mellitus by age group.

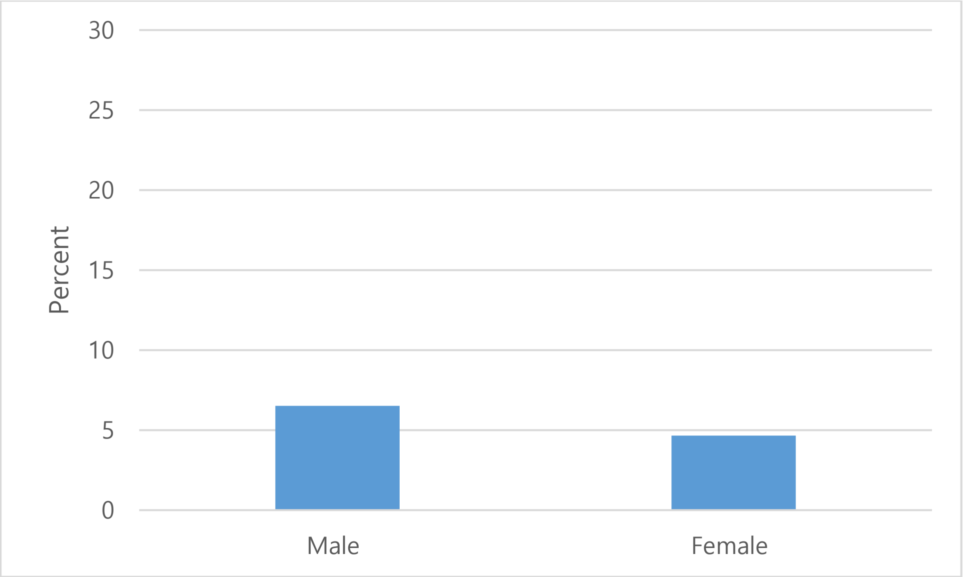

Figure S3. Proportion of patients with hypertension by gender.

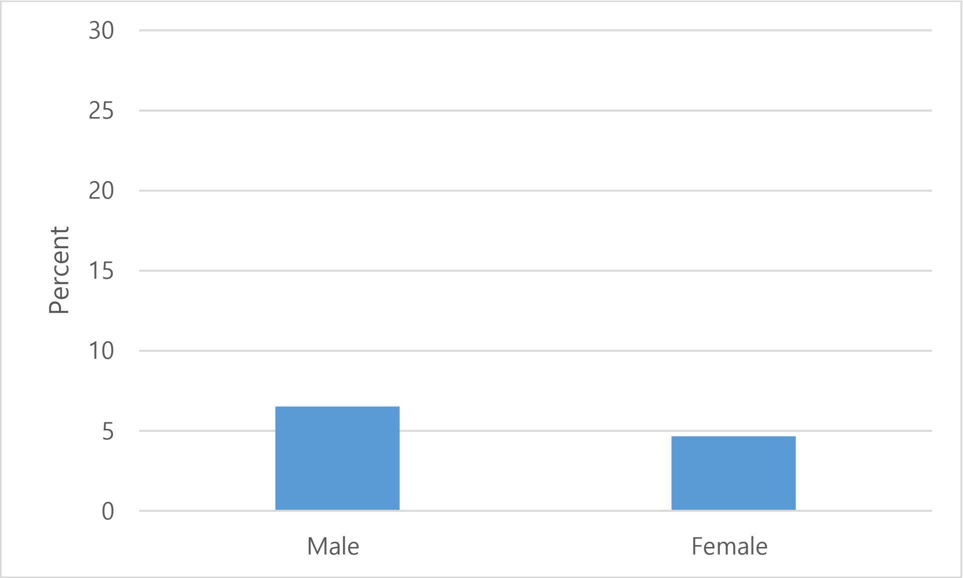

Figure S4. Proportion of patients with diabetes mellitus by gender.
